# Root resource foraging: does it matter?

**DOI:** 10.3389/fpls.2013.00303

**Published:** 2013-08-12

**Authors:** Xin Tian, Peter Doerner

**Affiliations:** ^1^Institute for Molecular Plant Science, School of Biological Sciences, University of EdinburghEdinburgh, Scotland; ^2^Laboratoire de Physiologie Cellulaire Végétale, CNRS, CEA, INRA, Université Grenoble AlpesGrenoble, France

Root traits have recently come out of the dark to take center stage in efforts to increase food production and transition toward sustainability, with calls for a “Second Green Revolution” focused on roots (Lynch, 2007; Den Herder et al., 2010). We will highlight some of the challenges *en route* to developing fruitful strategies for enhanced resource acquisition in crops. The article will focus on resource foraging, the process by which root system architecture (RSA, the ensemble of the root system in space) changes over time to acquire resources. Most studies on foraging in plants have focused on resource competition between wild species, using plants subjected to natural selection, not to agricultural selection (Cahill and McNickle, 2011). These studies have been extremely useful to begin to identify the mechanisms and selective forces that shape adaptation and survival in natural environments (Kembel et al., 2008; McNickle et al., 2009; de Kroon et al., 2012; Craine and Dybzinski, 2013; Padilla et al., 2013). A striking, albeit not surprising, conclusion from these studies is the diversity of root behavior, including foraging, observed in different species, even in different accessions of the same species, revealing its adaptive nature.

## What is resource foraging?

The Oxford Dictionary defines foraging as: “a wide search over an area in order to obtain something, especially food or provisions.” For many animals, foraging is a motile behavior essential for survival. To survive, sessile plants must acquire resources that are differentially and non-uniformly distributed in space and time: Nitrate is relatively mobile in most soils—hence will percolate into deeper layers with water, and be more evenly distributed; by contrast, inorganic phosphate is relatively immobile, will distribute heterogeneously, in fertile agricultural soils mostly in the surface layer (Marschner, 1995). Thus, when plants explore their environment there is no single, optimal strategy equally suited for all nutrients. Root behavior can be defined as the tropisms and growth activities that vary quantitatively over time, such as changes to growth rate (of each meristem), direction (angle, tortuosity), and root density (rate of lateral root primordia formation and emergence, and demography of lateral root meristems per unit cell number of lower order root). While each of these parameters can impact foraging capacity, those that affect the behavior of lateral organs stand out, because they define the capacity to exploit locally enriched resources.

## How do plants forage for resources?

Foraging in animals is defined as “searching” for resources—do plant roots *prospect* for resources, that is grow without prior perception of resources; or does the *perception* of resources promote growth behaviors that facilitate their exploitation? This distinction is not semantic, because targeted strategies to improve resource acquisition would focus on different processes.

Recent work in Arabidopsis has shown that lateral root primordia (LRP) are formed in a strictly acropetal sequence, with no new LRP formed between already developed lateral roots (LR) or LRP (Dubrovsky et al., 2000, 2001, 2006; Moreno-Risueno et al., 2010). However, not every LRP develops into an LR (Zhang et al., 1999; Dubrovsky et al., 2006), indicating the presence of a facultative checkpoint, which may be responsive to developmental, environmental or physiologic cues. Note that these experiments were performed in artificial, axenic conditions: synthetic media with uniform nutrient distribution at the onset of the experiment and the absence of any root-microbe interactions, or of obstacles, whose mechanical impedance affects growth behavior (Fang et al., 2013). Therefore, their significance for root systems in more natural environments remains to be confirmed. However, precisely these conditions permitted a very rigorous, detailed analysis of the initiation and early development of LRP, which is challenging in a soil environment. Despite these caveats, the findings of these and other studies suggest that resource perception stimulates outgrowth, of at least a fraction of LRP in response to environmental cues. Resource perception likely occurs through processes localized in the root cap, which is involved in the perception and orchestration of responses to several environmental cues (Eapen et al., 2005; Svistoonoff et al., 2007; Baldwin et al., 2013), and which is consistent with a requirement for pre-existing LRP to be activated in the vicinity of resource patches, rather than *de novo* production of primordia.

Root growth behavior is very variable (“plasticity”), irrespective of the growth substrate. Several studies have shown that for example, growth rates of individual lateral roots, even in “homogeneous” environments, vary greatly (Pages, 1995; Freixes et al., 2002; Forde, 2009). Thus, the magnitude of growth activities is not simply proportional to resources in the environment, which seems to contradict the earlier conclusion that perception of resources promotes growth behaviors that facilitate their exploitation. Such growth variability has been termed “developmental noise” to highlight its stochastic nature, and it has been argued that the contribution of developmental noise to the overall elaboration of the root system exceeds that of resource-responsive modulation of LR growth (Forde, 2009).

In a classical set of experiments, Drew and collaborators showed that barley root system growth responds differently to similar environmental nutrient concentrations, dependent on resource distribution. When nutrients were uniformly distributed throughout the root's environment, LR growth was not greatly stimulated, whereas when heterogeneously distributed, root growth in the vicinity of high-nutrient patches was significantly enhanced (Drew, 1975; Drew and Saker, 1975). These distinct responses appear adaptively advantageous, when one considers that plants need to invest previously assimilated limiting resources, such as photosynthate and amino acids, to grow roots. Earlier work has shown a strong correlation between the hexose concentration in individual LR meristems and their growth rate (Freixes et al., 2002); strong demand for and consumption of hexoses in active meristems reinforces local phloem unloading, which ensures elevated assimilate provision to active meristems. Moreover, it was recently shown that soluble carbohydrates (sucrose, glucose) also stimulate auxin biosynthesis in a dose-dependent manner (Sairanen et al., 2012). Together, these observations imply the existence of positive feedback mechanisms that reinforce the outcome of competition between individual LRP and LR meristems for systemic resources within the plant. Such mechanisms to limit the number of active LR meristems may largely underpin the phenomenon of “developmental noise.”

In addition to the factors controlling root growth rate and density, other aspects also play important roles in a root system's capacity to acquire resources: growth angle and tortuosity, as well as root hairs. The former parameters are particularly important in domesticated plants in an agricultural context, because managed soil environments generally have higher nutrient levels in the surface layer, partly caused by fertilizer provision. Thus, unsurprisingly, modern barley cultivars not only have different RSA than their wild progenitors or landraces, furthermore, the growth angles of their nodal roots (which grow close to the surface) are shallower (Grando and Ceccarelli, 1995; de Dorlodot et al., 2007). Tortuosity, the ratio between the actual length of the root and the shortest distance between its origin and tip (Tracy et al., 2012), which is a surrogate measure for the extent to which a soil volume is sampled for nutrients, may not only be the outcome of obstacle avoidance, but could be part of a foraging strategy for immobile nutrients. Recently, it was shown that some root growth-regulatory peptides enhance tortuosity by interfering with auxin-dependent control of root growth direction (Whitford et al., 2012; Fernandez et al., 2013), which may be exploited in the future to enhance nutrient capture. Root hairs are important to extend the reach of roots radially; their length responds to the availability of immobile resources such as iron or phosphate (Schmidt and Schikora, 2001; Müller and Schmidt, 2004).

## Resource capture in non-domesticated plants

Studies in wild plants have revealed a remarkable level of RSA variability, shaped by inter- and intra-species competition in plant communities for limiting resources (Cahill and McNickle, 2011). These studies showed that many plants sense, and can distinguish, the presence of kin and non-kin plants, and can adjust RSA responses correspondingly. Competition and survival under limiting and non-uniformly distributed resource availability have selected for not necessarily the most energy-efficient strategies (maximizing resource acquisition with minimal energy input), but rather aim at resource pre-emption (super-optimal resource acquisition to negate their availability to potential competitors) (Craine et al., 2005; McNickle et al., 2009; McNickle and Cahill, 2009; Cahill et al., 2010; Cahill and McNickle, 2011; Nord et al., 2011). It is not yet clear, whether that strategy is universal; if it were and applicable to domesticated crops, this would point to a future bottleneck in crop improvement efforts, for which optimizing the plant's energy expenditure in relation to resource acquisition would be important.

## Is increased foraging capacity in crops desirable?

Although agricultural practice in rich countries is characterized by regular inputs, particularly in the form of high-level fertilization and irrigation, this practice is not sustainable (Foley et al., 2011; Mueller et al., 2012). While increasing foraging capacity and efficiency in crops would not bypass the requirement to supplement resources, it will allow crops to utilize soil resources more extensively and efficiently, reduce resource losses due to environmental run-off, and, most importantly, facilitate the transition to productive agro-ecosystems with low inputs. The largest impact of this would be felt where it matters most - in those countries where farmers cannot afford high inputs. Improving foraging capacity would also aim to increase resource acquisition efficiency (RAE), although it is not certain that it would simultaneously increase resource utilization efficiency (RUE), which is the efficiency with which a unit of resource is converted into (harvestable) biomass (Rose and Wissuwa, 2012).

## Conclusions

Despite many recent advances in our understanding of the genetic and mechanistic control of root growth, we are still very far from an operational understanding of the genetic mechanisms that underpin root system behavior. Recent technological advances, such as X-ray tomography, will without question facilitate progress by enabling a much more detailed understanding of the spatial and temporal aspects of root system growth (Mairhofer et al., 2012; Tracy et al., 2012). Further technological breakthroughs are needed, particularly in the analysis of RSA when multiple plants, kin or non-kin, are growing together to better understand the conditional interaction of homo- and heterologous root systems in different soil environments, as this will be important for the development of future cropping systems and the suitability of specific cultivars.

Resource acquisition in roots is a complex trait, governing root growth behavior and acquisition by transporters and assimilation into metabolic pathways. Therefore it is dependent on allelic-specific interactions of many genes, although some cases of single, large effect quantitative trait loci (QTL) underpinning improved performance are known (Gamuyao et al., 2012). Targeted approaches based on single-gene based transgenic approaches are necessary, but unlikely to be sufficient or deliver results in time to provide a doubling of food production by 2050 (The Royal Society, 2009). Therefore, it is an urgent priority to exploit the natural variants selected for over centuries in landraces or wild relatives of crops that perform well in soils with poor resources (Lynch, 2007), to provide material for breeding programs that combine yield traits from elite varieties with resource traits from landraces or wild accessions. Such breeding programs have the potential to rapidly deliver results, particularly where it matters most: in poor countries, to markedly enhance yields in low-input agricultural systems. This should be a priority, as investments here will yield the greatest initial returns, together with the highest likelihood of rapid adoption.

To secure sustainable food production, it is imperative to pursue both fundamental research into root growth mechanisms to inform transgenic approaches *and* breeding programs based on existing germplasm. Since sustainable agricultural practices aim to transform current high-input monoculture-based agro-ecosystems into ecosystems more similar to natural environments, with lower inputs and multiple or mixed cropping systems, research into root foraging should not be restricted to crop plants and their interactions. We have much to learn from wild plants how inter-species competition and different niches have shaped root foraging strategies in evolution in the quest for more resilient performance for crops experiencing environmental stresses.

## References

[B1] Baldwin K. L. Strohm A. K. Masson P. H. (2013). Gravity sensing and signal transduction in vascular plant primary roots. Am. J. Bot. 100, 126–142 10.3732/ajb.1200318 23048015

[B2] Cahill J. F. McNickle G. G. (2011). The behavioral ecology of nutrient foraging by plants. Annu. Rev. Ecol. Evol. Syst. 42, 289–311 10.1146/annurev-ecolsys-102710-145006

[B3] Cahill J. F. McNickle G. G. Haag J. J. Lamb E. G. Nyanumba S. M. Clair C. C. S. (2010). Plants integrate information about nutrients and neighbors. Science 328, 1657–1657 10.1126/science.1189736 20576883

[B4] Craine J. M. Dybzinski R. (2013). Mechanisms of plant competition for nutrients, water and light. Funct. Ecol. 27, 833–840 10.1111/1365-2435.12081

[B5] Craine J. M. Fargione J. Sugita S. (2005). Supply pre-emption, not concentration reduction, is the mechanism of competition for nutrients. New Phytol. 166, 933–940 10.1111/j.1469-8137.2005.01386.x 15869653

[B6] de Dorlodot S. Forster B. Pages L. Price A. Tuberosa R. Draye X. (2007). Root system architecture: opportunities and constraints for genetic improvement of crops. Trends Plant Sci. 12, 474–481 10.1016/j.tplants.2007.08.012 17822944

[B7] de Kroon H. Hendriks M. Van Ruijven J. Ravenek J. Padilla F. M. Jongejans E. (2012). Root responses to nutrients and soil biota: drivers of species coexistence and ecosystem productivity. J. Ecol. 100, 6–15 10.1111/j.1365-2745.2011.01906.x

[B8] Den Herder G. Van Isterdael G. Beeckman T. De Smet I. (2010). The roots of a new green revolution. Trends Plant Sci. 15, 600–607 10.1016/j.tplants.2010.08.009 20851036

[B9] Drew M. C. (1975). Comparison of effects of a localized supply of phosphate, nitrate, ammonium and potassium on growth of seminal root system, and shoot, in barley. New Phytol. 75, 479–490 10.1111/j.1469-8137.1975.tb01409.x

[B10] Drew M. C. Saker L. R. (1975). Nutrient supply and growth of seminal root system in barley.2. Localized, compensatory increases in lateral root growth and rates of nitrate uptake when nitrate supply is restricted to only part of root system. J. Exp. Bot. 26, 79–90 10.1093/jxb/26.1.79

[B11] Dubrovsky J. G. Doerner P. W. Colon-Carmona A. Rost T. L. (2000). Pericycle cell proliferation and lateral root initiation in Arabidopsis. Plant Physiol. 124, 1648–1657 10.1104/pp.124.4.1648 11115882 PMC59863

[B12] Dubrovsky J. G. Gambetta G. A. Hernandez-Barrera A. Shishkova S. Gonzalez I. (2006). Lateral root initiation in Arabidopsis: developmental window, spatial patterning, density and predictability. Ann. Bot. 97, 903–915 10.1093/aob/mcj604 16390845 PMC2803408

[B13] Dubrovsky J. G. Rost T. L. Colon-Carmona A. Doerner P. (2001). Early primordium morphogenesis during lateral root initiation in *Arabidopsis thaliana*. Planta 214, 30–36 10.1007/s004250100598 11762168

[B14] Eapen D. Barroso M. L. Ponce G. Campos M. E. Cassab G. I. (2005). Hydrotropism: root growth responses to water. Trends Plant Sci. 10, 44–50 10.1016/j.tplants.2004.11.004 15642523

[B15] Fang S. Clark R. T. Zheng Y. Iyer-Pascuzzi A. S. Weitz J. S. Kochian L. V. (2013). Genotypic recognition and spatial responses by rice roots. Proc. Natl. Acad. Sci. U.S.A. 110, 2670–2675 10.1073/pnas.1222821110 23362379 PMC3574932

[B16] Fernandez A. Drozdzecki A. Hoogewijs K. Nguyen A. Beeckman T. Madder A. (2013). Transcriptional and functional classification of the GOLVEN/ROOT GROWTH FACTOR/CLE-like signaling peptides reveals their role in lateral root and hair formation. Plant Physiol. 161, 954–970 10.1104/pp.112.206029 23370719 PMC3561032

[B17] Foley J. A. Ramankutty N. Brauman K. A. Cassidy E. S. Gerber J. S. Johnston M. (2011). Solutions for a cultivated planet. Nature 478, 337–342 10.1038/nature10452 21993620

[B18] Forde B. G. (2009). Is it good noise? the role of developmental instability in the shaping of a root system. J. Exp. Bot. 60, 3989–4002 10.1093/jxb/erp265 19759097

[B19] Freixes S. Thibaud M. C. Tardieu F. Muller B. (2002). Root elongation and branching is related to local hexose concentration in *Arabidopsis thaliana* seedlings. Plant Cell Environ. 25, 1357–1366 10.1046/j.1365-3040.2002.00912.x

[B20] Gamuyao R. Chin J. H. Pariasca-Tanaka J. Pesaresi P. Catausan S. Dalid C. (2012). The protein kinase Pstol1 from traditional rice confers tolerance of phosphorus deficiency. Nature 488, 535–539 10.1038/nature11346 22914168

[B21] Grando S. Ceccarelli S. (1995). Seminal root morphology and coleoptile length in wild (*Hordeum vulgare* ssp. spontaneum) and cultivated (*Hordeum vulgare* ssp. vulgare) barley. Euphytica 86, 73–80 10.1007/BF0003594123124391

[B22] Kembel S. W. de Kroon H. Cahill J. F. Mommer L. (2008). Improving the scale and precision of hypotheses to explain root foraging ability. Ann. Bot. 101, 1295–1301 10.1093/aob/mcn044 18424813 PMC2710254

[B23] Lynch J. P. (2007). Roots of the second green revolution. Aust. J. Bot. 55, 493–512 10.1071/BT061189120625

[B24] Mairhofer S. Zappala S. Tracy S. R. Sturrock C. Bennett M. Mooney S. J. (2012). RooTrak: automated recovery of three-dimensional plant root architecture in soil from x-ray microcomputed tomography images using visual tracking. Plant Physiol. 158, 561–569 10.1104/pp.111.186221 22190339 PMC3271750

[B25] Marschner H. (1995). Mineral Nutrition of Higher Plants. London: Academic Press.

[B26] McNickle G. G. Cahill J. F. (2009). Plant root growth and the marginal value theorem. Proc. Nat. Acad. Sci. U.S.A. 106, 4747–4751 10.1073/pnas.0807971106 19264964 PMC2660770

[B27] McNickle G. G. St Clair C. C. Cahill J. F. (2009). Focusing the metaphor: plant root foraging behaviour. Trends Ecol. Evol. 24, 419–426 10.1016/j.tree.2009.03.004 19409652

[B28] Moreno-Risueno M. A. Van Norman J. M. Moreno A. Zhang J. Ahnert S. E. Benfey P. N. (2010). Oscillating gene expression determines competence for periodic Arabidopsis root branching. Science 329, 1306–1311 10.1126/science.1191937 20829477 PMC2976612

[B29] Mueller N. D. Gerber J. S. Johnston M. Ray D. K. Ramankutty N. Foley J. A. (2012). Closing yield gaps through nutrient and water management. Nature 490, 254–257 10.1038/nature11420 22932270

[B30] Müller M. Schmidt W. (2004). Environmentally induced plasticity of root hair development in Arabidopsis. Plant Physiol. 134, 409–419 10.1104/pp.103.029066 14730071 PMC371035

[B31] Nord E. A. Zhang C. C. Lynch J. P. (2011). Root responses to neighbouring plants in common bean are mediated by nutrient concentration rather than self/non-self recognition. Funct. Plant Biol. 38, 941–952 10.1071/FP11130 32480953

[B32] Padilla F. M. Mommer L. de Caluwe H. Smit-Tiekstra A. E. Wagemaker C.a.M. Ouborg N. J. (2013). Early root overproduction not triggered by nutrients decisive for competitive success belowground. PLoS ONE 8:e55805 10.1371/journal.pone.0055805 23383284 PMC3561313

[B33] Pages L. (1995). Growth-patterns of the lateral roots of young oak (*Quercus-Robur*) tree seedlings - relationship with apical diameter. New Phytol. 130, 503–509 10.1111/j.1469-8137.1995.tb04327.x 33874478

[B34] Rose T. J. Wissuwa M. (2012). Rethinking internal phosphorus utilization efficiency: a new approach is needed to improve pue in grain crops. Adv. Agron. 116, 185–217 10.1016/B978-0-12-394277-7.00005-1

[B35] Sairanen I. Novak O. Pencik A. Ikeda Y. Jones B. Sandberg G. (2012). Soluble carbohydrates regulate auxin biosynthesis via PIF proteins in Arabidopsis. Plant Cell 24, 4907–4916 10.1105/tpc.112.104794 23209113 PMC3556965

[B36] Schmidt W. Schikora A. (2001). Different pathways are involved in phosphate and iron stress-induced alterations of root epidermal cell development. Plant Physiol. 125, 2078–2084 10.1104/pp.125.4.2078 11299387 PMC88863

[B37] The Royal Society. (2009). Reaping the Benefits: Science and the Sustainable Intensification of Global Agriculture. London: The Royal Society.

[B38] Svistoonoff S. Creff A. Reymond M. Sigoillot-Claude C. Ricaud L. Blanchet A. (2007). Root tip contact with low-phosphate media reprograms plant root architecture. Nat. Genet. 39, 792–796 10.1038/ng2041 17496893

[B39] Tracy S. R. Black C. R. Roberts J. A. Sturrock C. Mairhofer S. Craigon J. (2012). Quantifying the impact of soil compaction on root system architecture in tomato (*Solanum lycopersicum*) by x-ray micro-computed tomography. Ann. Bot. 110, 511–519 10.1093/aob/mcs031 22362666 PMC3394635

[B40] Whitford R. Fernandez A. Tejos R. Perez A. C. Kleine-Vehn J. Vanneste S. (2012). GOLVEN secretory peptides regulate auxin carrier turnover during plant gravitropic responses. Dev. Cell 22, 678–685 10.1016/j.devcel.2012.02.002 22421050

[B41] Zhang H. Jennings A. Barlow P. W. Forde B. G. (1999). Dual pathways for regulation of root branching by nitrate. Proc. Natl. Acad. Sci. U.S.A. 96, 6529–6534 10.1073/pnas.96.11.6529 10339622 PMC26916

